# Serum from patients with chronic obstructive pulmonary disease induces senescence-related phenotype in bronchial epithelial cells

**DOI:** 10.1038/s41598-018-31037-w

**Published:** 2018-08-28

**Authors:** Barbara Kuźnar-Kamińska, Justyna Mikuła-Pietrasik, Anna Witucka, Aleksandra Romaniuk, Natalia Konieczna, Błażej Rubiś, Krzysztof Książek, Andrzej Tykarski, Halina Batura-Gabryel

**Affiliations:** 1 Department of Pulmonology, Allergology and Respiratory Oncology, Poznań University of Medical Sciences, Szamarzewskiego 84 Str., 60-569 Poznań, Poland; 2 Department of Hypertensiology, Angiology and Internal Medicine, Poznań University of Medical Sciences, Długa 1/2 Str., 61-848 Poznań, Poland; 3 Department of Clinical Chemistry and Molecular Diagnostics, Poznan University of Medical Sciences, Przybyszewskiego 49 Str., 60-355 Poznań, Poland

## Abstract

Chronic obstructive pulmonary disease (COPD) is a risk factor for the development of lung cancer (LC). The mechanism of interplay between both diseases remains poorly recognized. This report examines whether COPD may cause a senescence response in human bronchial epithelial cells (HBECs), leading to the progression of LC in a senescence-dependent manner. The results show that HBECs exposed to serum from COPD patients manifest increased expression of markers of cellular senescence, including senescence-associated β-galactosidase (SA-β-Gal), histone γ-H2A.X, and p21, as compared to the serum of healthy donors. This effect coincides with an increased generation of reactive oxygen species by these cells. The clinical analysis demonstrated that COPD may cause the senescence, independently on smoking status and disease severity. The concentrations of CXCL5, CXCL8/IL-8 and VEGF were higher in conditioned medium (CM) harvested from HBECs after exposure to COPD serum as compared to controls. In addition, CM treated with serum from COPD patients stimulated adhesion of A549 cancer cells to HBECs, as well as accelerating cancer cell proliferation and migration *in vitro*. Collectively, these findings indicate that COPD may induce senescence-like changes in HBECs and thus enhance some processes associated with the progression of lung cancer.

## Introduction

Recent evidence suggests that some lung pathologies caused by environmental factors may be related to the development of cellular senescence^1,2^.

Chronic obstructive pulmonary disease (COPD) is a chronic pathology of the lungs mainly caused by tobacco smoking^3^. Individual studies have confirmed the participation of cellular senescence in the development of COPD, but its meaning in obstructive disease has not yet been completely elucidated^4–6^.

At the same time, it has been demonstrated that cellular senescence plays an important role in the progression of primary and metastatic tumors. Carcinogenic activity of senescent cells has been confirmed in the invasion of breast, ovarian, colorectal, pancreatic, and other cancers^7–10^.

COPD is a major risk factor for lung cancer, irrespective of nicotine use. Patients with obstructive disease develop lung tumors two to four times more frequently than do healthy smokers^11^. 23% of lung cancer patients were previously diagnosed with COPD^12^. The coexistence of both diseases, as well as our knowledge of the potential role of cellular senescence in the pathogenesis of COPD and lung cancer, is suggestive of their joint pathogenesis. Its importance in patients with both diseases has not yet been examined.

It is thus reasonable to determine whether COPD may elicit a senescence response in human bronchial epithelial cells (HBECs), leading implicitly to the progression of lung cancer in a senescence-dependent mechanism. To this end, we treated epithelial cells with serum from COPD patients, a fluid, which in line with our previous observations, may play a significant role in the COPD-related pathophysiology of lung cancer^13^. The choice of serum as a model of COPD was also based on the fact that an extravasation of blood proteins is an important element of the disease pathophysiology, actively contributing to the formation of a specific, pro-inflammatory milieu^14^. Last but not least, recent studies also suggest that serum may be a strong inducer of cellular senescence in various cell types, including glioblastoma cells and vascular endothelium^15,16^.

## Materials and Methods

### Chemicals

Unless otherwise stated, all chemicals and plastics were from Sigma (St. Louis, MO, USA). The tissue culture plastics were from Nunc (Roskilde, Denmark).

### Patients

The study was performed using serum samples obtained from COPD patients diagnosed according to the GOLD 2016 criteria, with all stages of airflow obstruction (1-mild, 2-moderate, 3-severe, 4-very severe) and all groups of the disease based on combined disease assessment using symptoms, spirometric classification and exacerbations history (A, B, C, D), both current and past smokers with a history of more than 20 pack-years, as well as nonsmokers (study group) and healthy volunteers (control group). Patients with stable disease status (without exacerbations in the six weeks prior to the study) were recruited during planned hospitalizations for COPD assessment at the Department of Pulmonology, Allergology, and Respiratory Oncology, or during planned visits to the Outpatient Clinic. The exclusion criteria were an inability to perform the spirometry tests, a history of asthma, tuberculosis, lung interstitial diseases, pulmonary thromboembolism, and other chronic pulmonary diseases. A control group was represented by current and past smokers with a history of more than 20 pack-years, as well as nonsmokers recruited among patients’ relatives and friends. All people potentially exposed to regular passive smoking were excluded. The exclusion criteria for the controls included any pulmonary disease.

The serum samples were taken in a fasting state and centrifuged immediately after collection. The serum was then stored in aliquots at −80 °C until required.

Clinical evaluation was performed after obtaining written informed consent, and with approval from the bioethics committee at Poznań University Ethics for research on human subjects (consent number 979/12). The methods were carried out in accordance with the relevant guidelines and regulations. The demographic data of the COPD patients and healthy volunteers (controls) are presented in Table 1.

**Table 1:** Characteristics of COPD patients and healthy volunteers (controls).

Parameter	COPD patients	Controls
N	100	60
Sex (female/male; n)	32/68	15/45
Age (mean ± SD/range; n)	64.88 ± 7.36/51–81	64.27 ± 8.49/49–83
Smoking (current/past/never; n)	39/57/4	23/18/19
Stage of obstruction (1/2/3/4)	6/15/42/37	N/A
COPD group (A/B/C/D)	9/6/13/72	N/A

### Cell culture and experimental conditions

Human bronchial epithelial cells (PromoCell, Germany) were cultured in a serum-free Airway Epithelial Cell Growth Medium, as recommended by the vendor. The medium was supplemented with bovine pituitary extract, epidermal growth factor, insulin, hydrocortisone, epinephrine, triiodo-L-thyronine, transferrin, and retinoic acid. The cells were seeded at low density onto the culture dishes and allowed to attach for 24 h. The cells were then exposed to 10% serum from COPD patients and healthy volunteers for 48 h. After incubation, the cells were subjected to further analyses. In some experiments, the cells exposed to the serum were carefully washed and subjected to a fresh, serum-free medium for 24 h to generate conditioned medium (CM) for immunoenzymatic assays. Some experiments were performed using lung cancer cell line A549. These cells were acquired from the American Type Culture Collection (Manassas, VA, USA) and propagated in Roswell Park Memorial Institute (RPMI)-1640 medium supplemented with 10% fetal bovine serum and antibiotics.

### Analysis of cellular senescence

The activity of senescence-associated β-galactosidase (SA-β-Gal), the presence of γ-H2A.X foci, and the expression of p21 cell cycle inhibitor were used to investigate the cellular senescence of the epithelial cells. SA-β-Gal activity was quantified in cell extracts by measuring the rate of conversion of 4-methylumbelliferyl-β-D-galactopyranose to 4-methylumbelliferone whereas the presence of SA-β-Gal-positive cells was visualized using a cytochemical reaction with X-gal, essentially as described in^17^. The expression of γ-H2A.X was quantified and visualized using an immunofluorescence-based method with a specific anti-γ-H2A.X antibody (Novus Biologicals, Littleton, USA), according to the protocol described in^9^. Representative pictures of SA-β-Gal and γ-H2A.X were taken using a Zeiss AxioVert.A1 (Carl Zeiss, Jena, Germany). The expression of p21 was examined using the p21 Waf1/Cip1 Total ELISA Kit (Invitrogen, Carlsbad, USA), as per the manufacturer’s instructions.

In some experiments, the bronchial epithelial cells were forced to replicative senescence by serial passaging until exhaustion of their proliferative capacity. The cells were considered senescent when they failed to divide within 4 consecutive weeks, displayed enlarged, hypertrophic morphology, and when >90% of them was stained positively for SA-β-Gal.

### Detection of reactive oxygen species (ROS)

ROS production by epithelial cells was assessed in cell extracts using 2′,7′-dichlorodihydrofluorescein diacetate (H_2_DCFDA), essentially as described in^18^.

### Immunoenzymatic assays

The concentration of VEGF, CXCL8, and CXCL5 in the CM generated by epithelial cells was quantified using appropriate DuoSet Immunoassay Development kits (R&D Systems, Wiesbaden, Germany), as per the manufacturer’s instructions.

### Measurement of cancer cell adhesion

Normal bronchial epithelial cells were placed in flat-bottom 96-well plates (1 × 10^3^) and left to settle. Afterwards, they were exposed to serum (10%, 48 h) from COPD patients and healthy volunteers. Cancer cells were detached by trypsinization, washed with phosphate-buffered saline (PBS), and probed with 5 µM calcein-AM (Molecular Probes, Invitrogen, Eugene, OR, USA) for 30 minutes at 37 °C. Calcein-labelled cells were washed with growth medium with 0.1% foetal bovine serum (FBS) to remove the free dye and added on top of normal bronchial epithelial cells. After 45 minutes of incubation at 37 °C, total fluorescence in each well was recorded using a spectrofluorimeter Synergy H1 (BioTek, Winooski, VT, USA) with 485 nm and 535 nm wavelengths for excitation and emission, respectively. Then, the non-adherent cells were removed by gentle washing and the measurement of fluorescence was repeated. To calculate the percentage of bound cells, the values recorded were compared with those representing the total fluorescence.

### Measurement of cancer cell proliferation

Cancer cell proliferation was examined using Cell Proliferation Kit I (PromoKine; Heidelberg, Germany). In brief, A549 lung cancer cells were seeded into culture dishes at a low-density (5 × 10^3^ cells per well), allowed to attach for 2 h, and then growth synchronized by serum deprivation for next 4 h. Afterwards, the cells were exposed to conditioned medium obtained from COPD patients and from healthy volunteers (10%, for 48 h). After the incubation, the cancer cells were probed with (5-(and 6)-carboxyfluorescein diacetate, succinimidyl ester, CFDA-SE (5 μM, for 15 minutes at 37 °C). Then, the cells were washed with culture medium and incubated for 5 min at 37 °C to hydrolyze free dye. Finally, the fluorescence of carboxyfluorescein, a product of the CFDA-SE transformation was recorded using the spectrofluorimeter Synergy H1 with 495 nm and 519 nm wavelengths for excitation and emission, respectively.

### Measurement of cancer cell migration

Cancer cell migration through a polycarbonate membrane (8 μM pores) towards the chemotactic gradient generated by conditioned medium produced by epithelial cells exposed to serum from COPD patients and healthy volunteers (10%, for 48 h) was examined using ChemoTx chambers (Neuro Probe, Gaithersburg, MD, USA). In brief, cancer cells were probed with calcein-AM (5 μM, 45 minutes, 37 °C), and then 4 × 10^4^ cells was suspended in serum-free growth medium and applied onto the top side of the filter to form a hemispherical drop. Afterward, the cells were allowed to migrate toward the conditioned media for 60 minutes at 37 °C. After the incubation, the membranes with cells that did not migrate were removed, and the intensity of fluorescence emitted by the cells that did migrate through the membranes was recorded using the spectrofluorimeter Synergy H1 with 485 nm and 535 nm wavelengths for excitation and emission, respectively.

### Statistics

The results were analyzed using GraphPad Prism v.5.00 software (GraphPad Software, San Diego, CA, USA). The groups were compared using the Mann–Whitney and Multiple comparisons were performed using Kruskal–Wallis analysis followed by Dunn’s Multiple Comparison post-test. Correlations were analyzed using the Spearman test. The results were expressed as means ± SEMs. Differences with a P value < 0.05 were considered to be statistically significant.

## Results and Discussion

Over the past few years, a body of evidence has arisen that cellular senescence plays an important role in the initiation and progression of lung cancer. It is intriguing that evidence is increasing that the same mechanisms are associated with COPD pathogenesis. Taking into account the frequent coexistence of these diseases, it seems necessary to take cellular senescence into consideration of the origin of lung cancer in COPD patients^19,20^.

Based on these facts, a project was designed to determine whether COPD can alter the phenotype of bronchial epithelial cells to a senescent-related. We decided to take HBECs for an experimental model because the majority of lung cancers are carcinomas that develop from the epithelial cells lining the airways^21^.

The experiments showed that serum harvested from the COPD patients causes bronchial epithelial cells to present features characteristic of a senescent phenotype.

**Figure 1:**
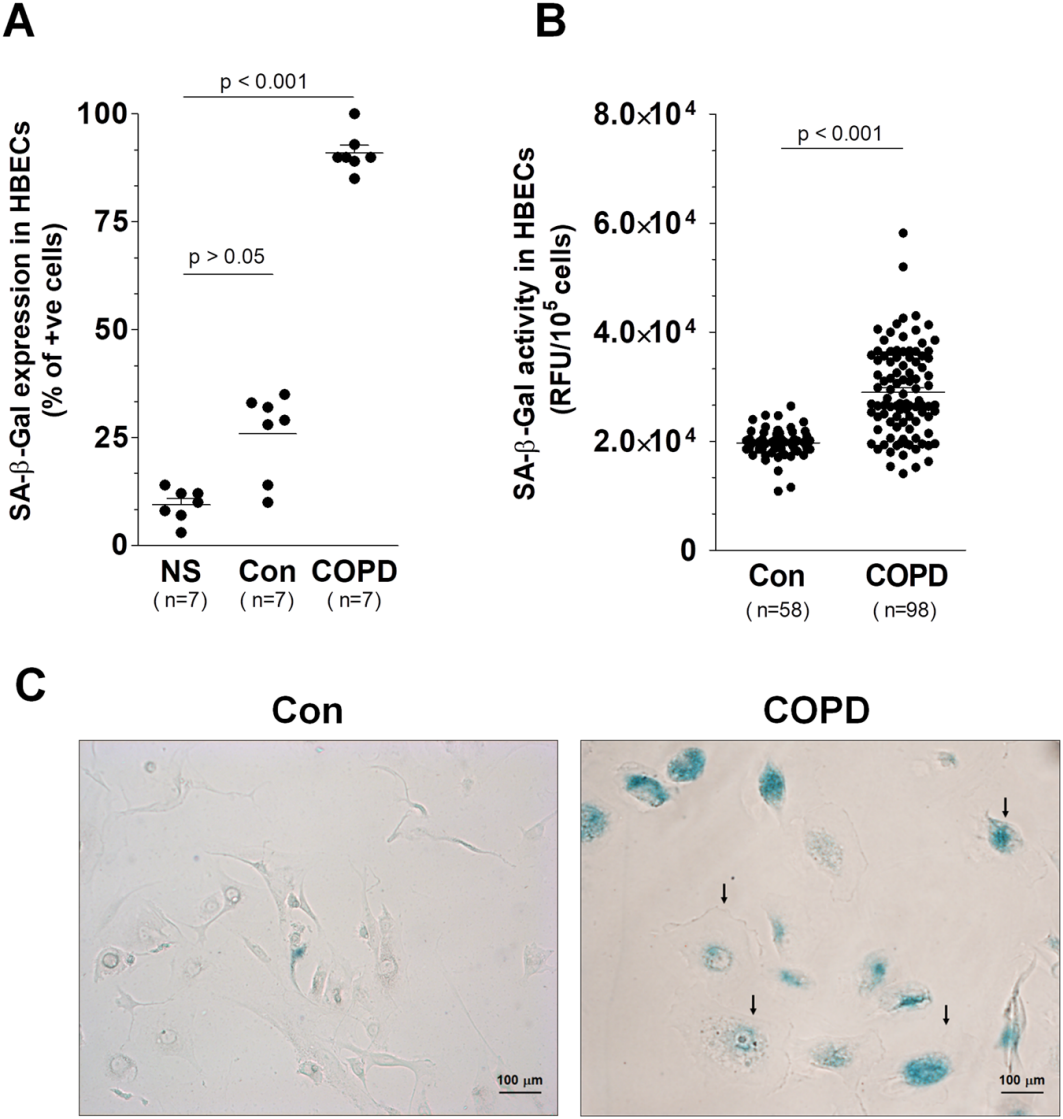
Effect of serum from COPD patients and healthy volunteers on: the percentage of SA-β-Gal positive cells (**A**) and the activity of SA-β-Gal (**B**). Panel (C) shows representative results of staining against SA-β-Gal (positive cells have green cytoplasm). The arrows indicate exemplary enlarged, hypertrophic, SA-β-Gal-positive cells. The number of positively stained cells was expressed as a percentage of 500 counted cells. The results are expressed as means ± SEM. The number of samples from each group is indicated in the brackets at the bottom of the graph. NS - nonstimulated cells; RFU - relative fluorescence units.

**Figure 2:**
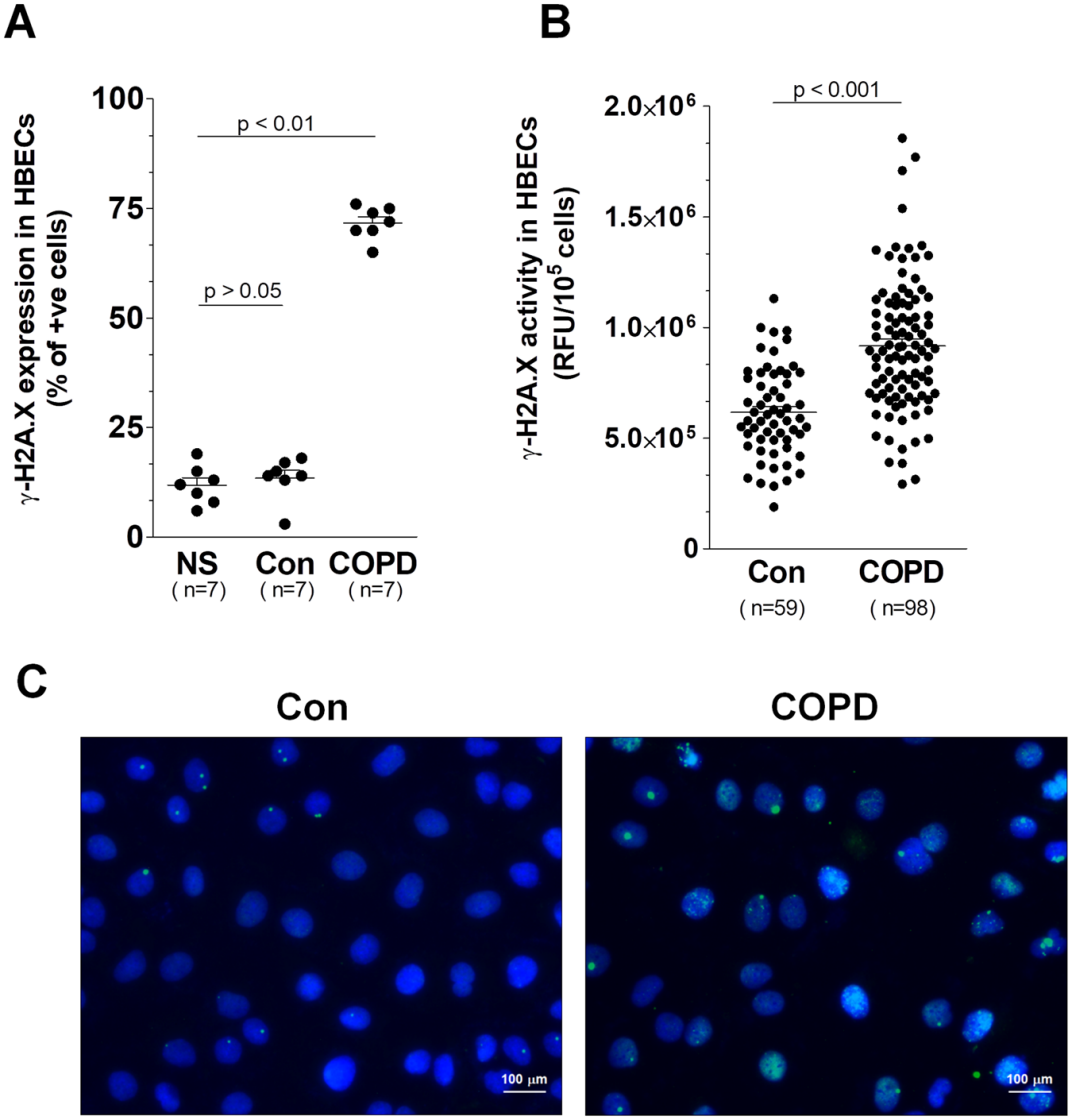
Effect of serum from COPD patients and healthy volunteers on the percentage of histone γ-H2A.X positive cells (**A**) and the activity of histone γ-H2A.X (**B**). Panel (C) shows representative results of staining against γ-H2A.X (positive cells display green foci within the nuclei marked with DAPI). The number of positively stained cells was expressed as a percentage of 500 counted cells. The number of samples from each group is indicated in the brackets at the bottom of the graph. The results are expressed as means ± SEM. NS - non-stimulated cells; RFU - relative fluorescence units.

Bronchial epithelial cells exposed to serum from COPD patients showed increased (vs. cells subjected to serum from healthy controls) expression of several markers of cellular senescence, including SA-β-Gal, histone γ-H2A.X, and cell cycle inhibitor, p21^22^. Importantly, as per the first two markers, their increased levels were demonstrated using both qualitative stainings (Figs 1A,C and 2A,C) and quantitative analysis (Figs 1B and 2B), which means that the serum from COPD patients really did increase the percentage of senescent epithelial cells in culture. We also analyzed the number of senescent cells before the epithelium exposure to both types of sera, finding that there was no significant impact of serum from healthy volunteers on the magnitude of cellular senescence.

**Figure 3:**
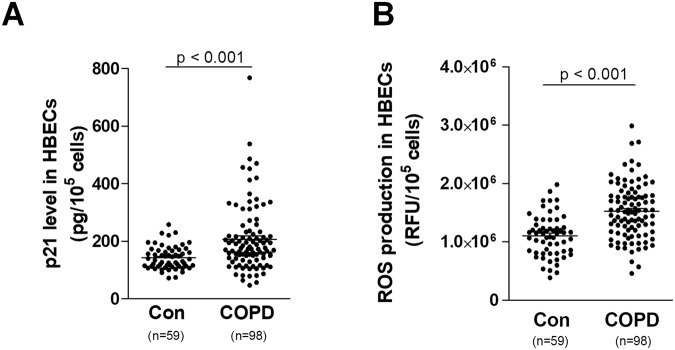
Effect of serum from COPD patients and healthy volunteers on the p21 level (**A**) and ROS production (**B**). The number of samples from each group is indicated in the brackets at the bottom of the graph. The results are expressed as means ± SEMs; RFU: relative fluorescence units.

In line with increased expression of p21 (Fig. 3A), this result may indicate that accelerated senescence of epithelial cells may proceed to some extent in a telomere-dependent mechanism, for which an up-regulated p21 is a significant effectory event^23^. This observation would seem to be important: in the classic (though probably simplistic) view, senescence of various kinds of epithelial cells proceeds mainly in a telomere-independent fashion^24^.

Analysis also revealed an increase in the production of ROS by the epithelial cells subjected to serum from COPD patients (Fig. 3B), which may suggest a causative role of oxidative stress in the premature senescence of the bronchial epithelium^25^. This finding is in line with results obtained on retinal pigment epithelial cells, whose senescence appeared to be accelerated by ROS, leading to an increased probability of the development of age-related macular degeneration^26^. The role of ROS in causing epithelial cell senescence has also been shown in HABECs exposed to nicotine^27^. This evidence is of special importance for understanding the molecular events that underlie the accelerated senescence of HABECs used in our experimental protocol, as the nicotine is the primary source of the cell and tissue abnormalities observed in patients with COPD^28^.

**Figure 4:**
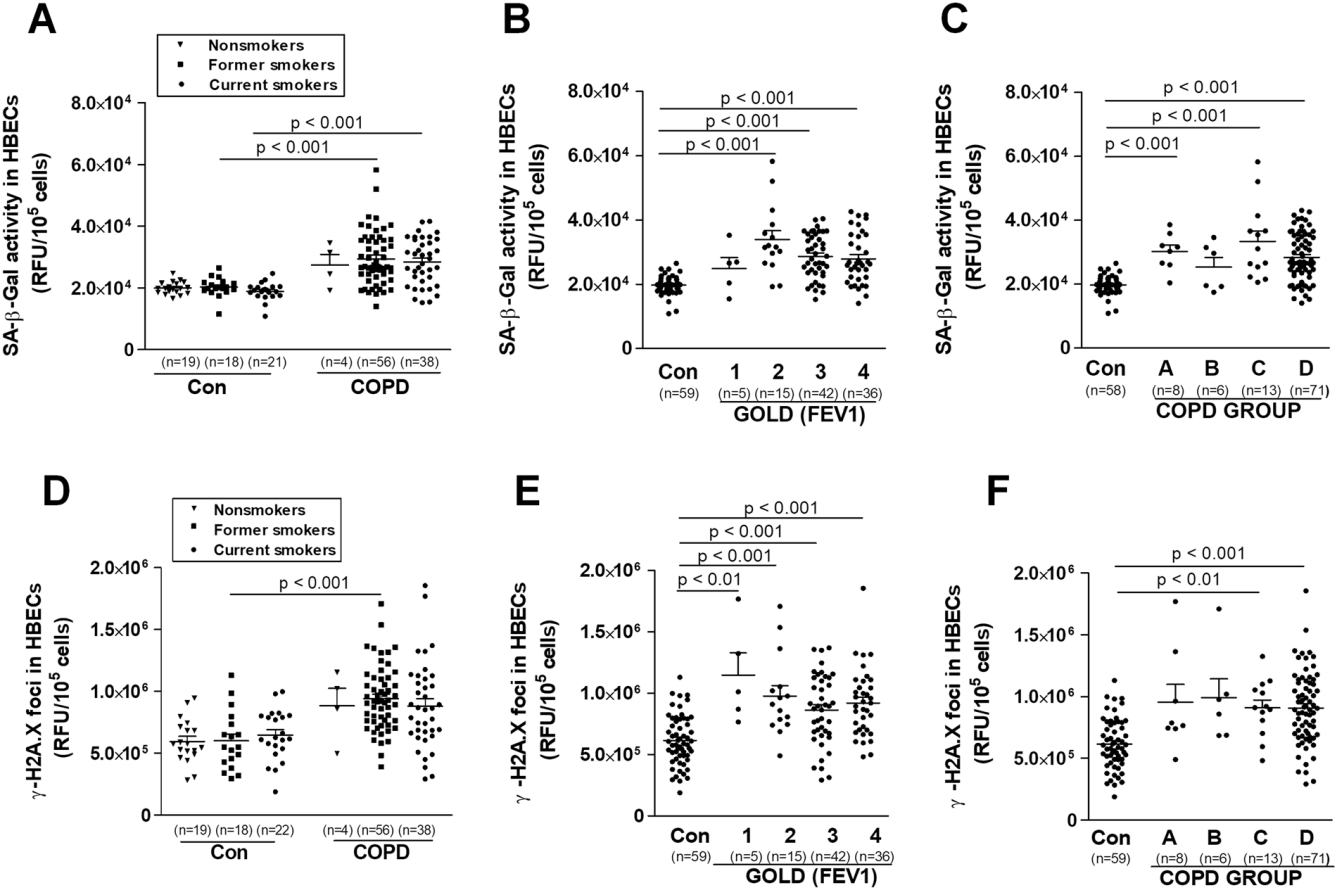
Effect of serum from COPD patients and healthy volunteers on the activity of SA-β-Gal (**A**–**C**) and the activity of histone γ-H2A.X (**D**–**F**) depending on smoking status, stage of obstruction, and COPD group, respectively. The number of samples from each group is indicated in the brackets at the bottom of the graph. The results are expressed as means ± SEMs. HBECs - human bronchial epithelial cells; RFU: relative fluorescence units.

**Figure 5:**
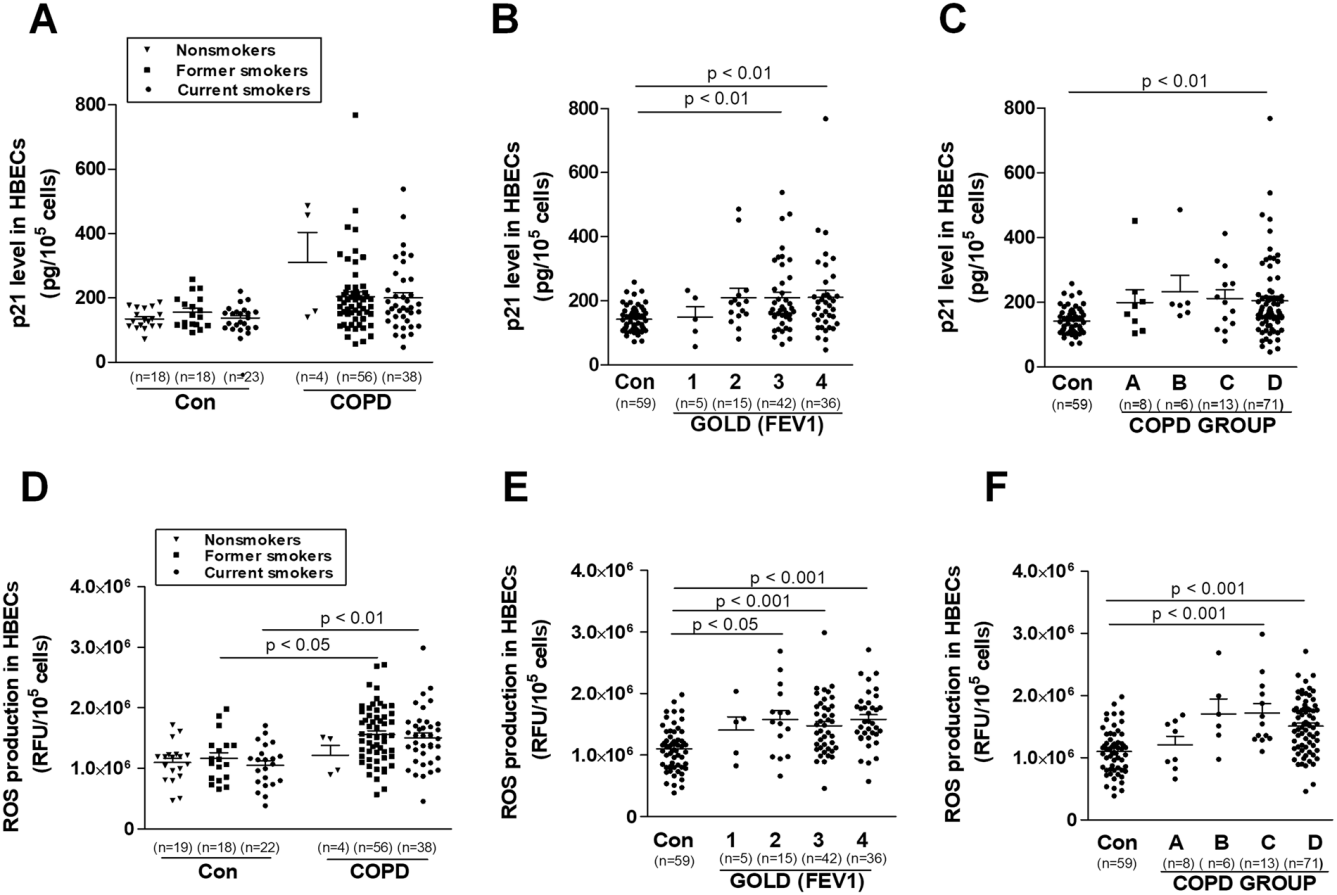
Effect of serum from COPD patients and healthy volunteers on the level of p21 (**A**–**C**) and the production of ROS (**D**–**F**) depending on smoking status, stage of obstruction, and COPD group, respectively. The number of samples from each group is indicated in the brackets at the bottom of the graph. The results are expressed as means ± SEMs; RFU: relative fluorescence units.

A significant part of this study involved reanalyzing of all four above-mentioned senescence-associated parameters from the point of view of the clinical status of the COPD patients from whom the serum was obtained (Figs 4 and 5). No effect of smoking status on senescence markers level was observed in COPD, as well as controls. When different smoking status COPD patients were compared to equal controls, levels of some senescence markers were higher for current smokers (SA-β-Gal and ROS) and former smokers (SA-β-Gal, γ-H2A.X, and ROS) (Figs 4A,D and 5A,D). Senescence markers levels didn’t differ between various stages of obstruction, as well as disease groups (Figs 4B,C,E,F and 5B,C,E,F). All analyzed senescence parameters didn’t correlate with FEV1 level. As cigarette smoke induces senescence in alveolar epithelial cells, we did not find in the literature any evidence that might verify senescence markers in various smoking COPD patients^29^. Tsuji *et al*. reported an increased percentage of p16 and p21 positive cells in type II alveolar cells of emphysematous patients as compared to healthy smokers and nonsmokers. Moreover, differently than in our study, both senescence markers negatively correlated with the predicted values of FEV1^5^. In the study of Kuwano *et al*., the decreased expression of SIRT6 (which may be related to the enhancement of cellular senescence in the IGF signaling pathway) was in line with severity of the obstruction^30^. The molecular considerations were supported by population studies confirming the increased risk of tumorigenesis in COPD patients in the most advanced stages of obstruction^31^. Our clinical observations reinforce hypothesis, that COPD may *per se* cause the senescence, independently on smoking status and disease severity.

**Figure 6:**
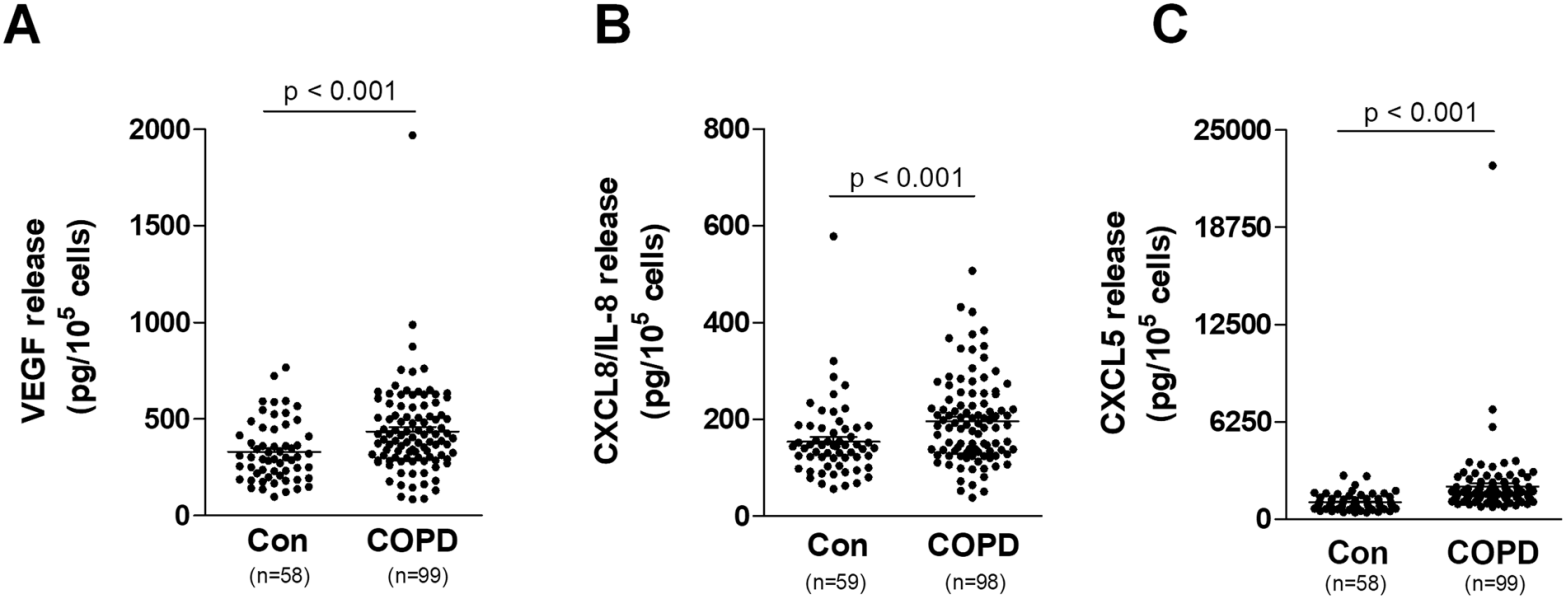
Effect of serum from COPD patients and healthy volunteers on the secretion of VEGF (**A**) CXCL8/IL-8 (**B**) and CXCL5 (**C**) by bronchial epithelial cells. The number of samples from each group is indicated in the brackets at the bottom of the graph. The results are expressed as means ± SEMs. RFU: relative fluorescence units.

**Figure 7:**
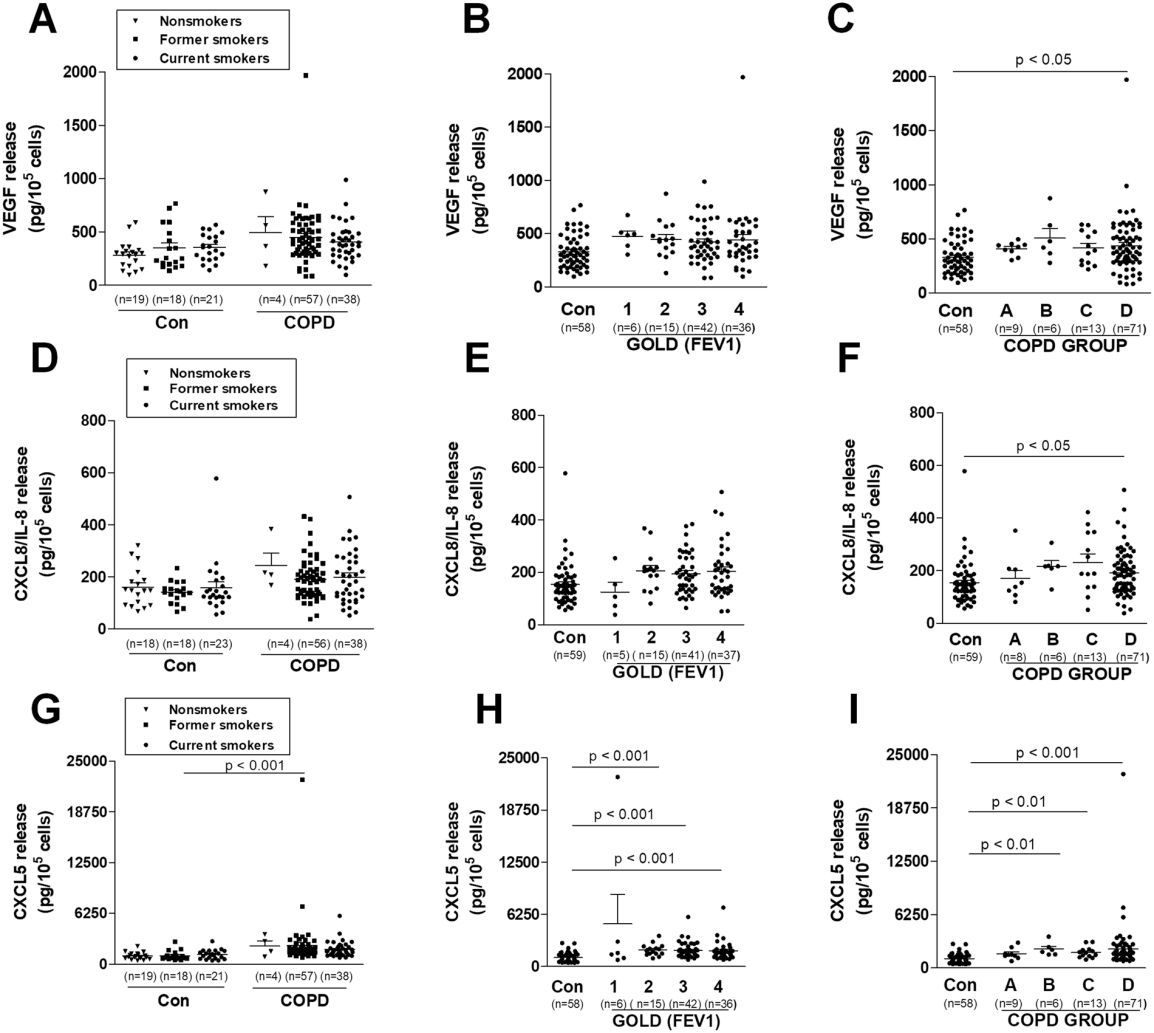
Effect of serum from COPD patients and healthy volunteers on the secretion of VEGF (**A**–**C**) CXCL8/IL-8 (**D**–**F**) and CXCL5 (**G**–**I**) depending on smoking status, stage of obstruction, and COPD group by bronchial epithelial cells, respectively. The number of samples from each group is indicated in the brackets at the bottom of the graph. The results are expressed as means ± SEMs. RFU: relative fluorescence units.

There is a broad consensus, that one of the most important signs of cellular senescence, mainly responsible for the contribution of senescent cells to several age-related pathologies, including cancer, is the so-called senescence-associated secretory phenotype (SASP)^10^. This refers to a situation where senescent cells release into the environment remarkably higher amounts of various proangiogenic, proinflammatory, and matrix remodeling factors than do their young, proliferating counterparts^32^. In this study we focused our attention on three proteins, VEGF, CXCL8/IL-8 and CXCL5, whose overproduction has already been described in the case of various types of senescent cells^33^. Here we showed that prematurely senescent bronchial epithelial cells maintained in the presence of serum from COPD patients display upregulated secretion of VEGF, CXCL8/IL-8 and CXCL5 (Fig. 6). No effects of smoking status, COPD stage, as well as COPD group on VEGF, CXCL8/IL-8 and CXCL5 levels were noticed (Fig. 7A–I).

The composition of SASP can differ depending on the type of senescent cell, thereby causing other consequences, including tumorigenesis, as has been confirmed by Coppe *et al*.^34^. A senescence-dependent increase has been observed in VEGF production in human and mouse fibroblasts in culture-stimulated vein endothelial cells that invaded a basement membrane^35^. In an earlier study, the CXCL5 level in the secretory profile was not changed at senescence, but the loss of p53 or gain of oncogenic RAS increases CXCL5 concentration^33^. CXCL5 indeed enhanced invasiveness and migration of breast cancer cells by upregulating fibroblast markers^36^. As in our study, the high level of CXCL8/IL-8 secreted by senescent fibroblasts in breast cancer enhanced the invasiveness of a panel of cancer cell lines (including epithelial cells) in cell-culture models^34^. The studies cited here are only individual examples supporting the progression of tumors through angiogenic markers secreted by senescent cells.

**Figure 8:**
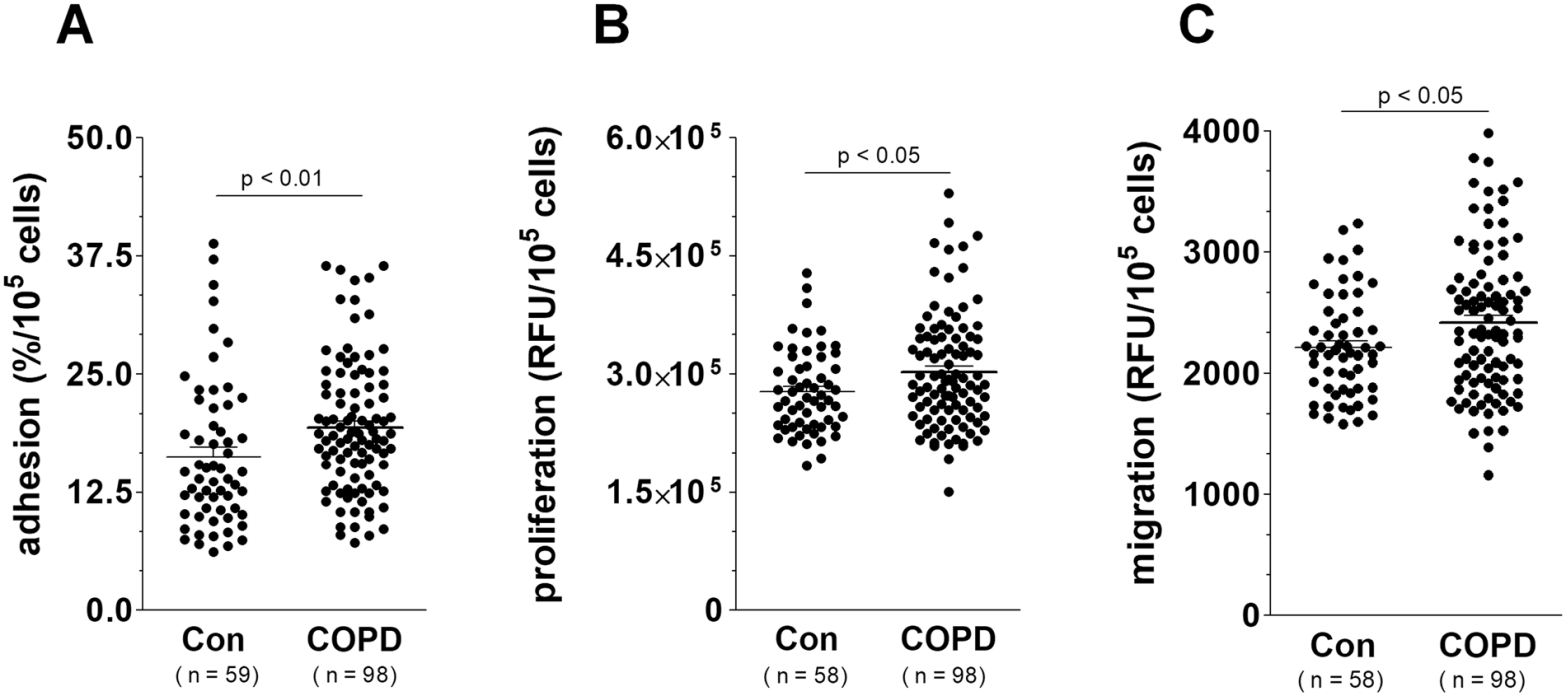
Effect of serum from COPD patients and healthy volunteers on adhesion (**A**) proliferation (**B**) and migration (**C**) of A549 lung cancer cells. Adhesion was tested in the presence of epithelial cells exposed to both types of serum. The proliferation and migration of cancer cells were analyzed through their exposure to conditioned medium generated by the epithelial cells subjected to the sera. The results are expressed as means ± SEMs. RFU: relative fluorescence units.

**Figure 9:**
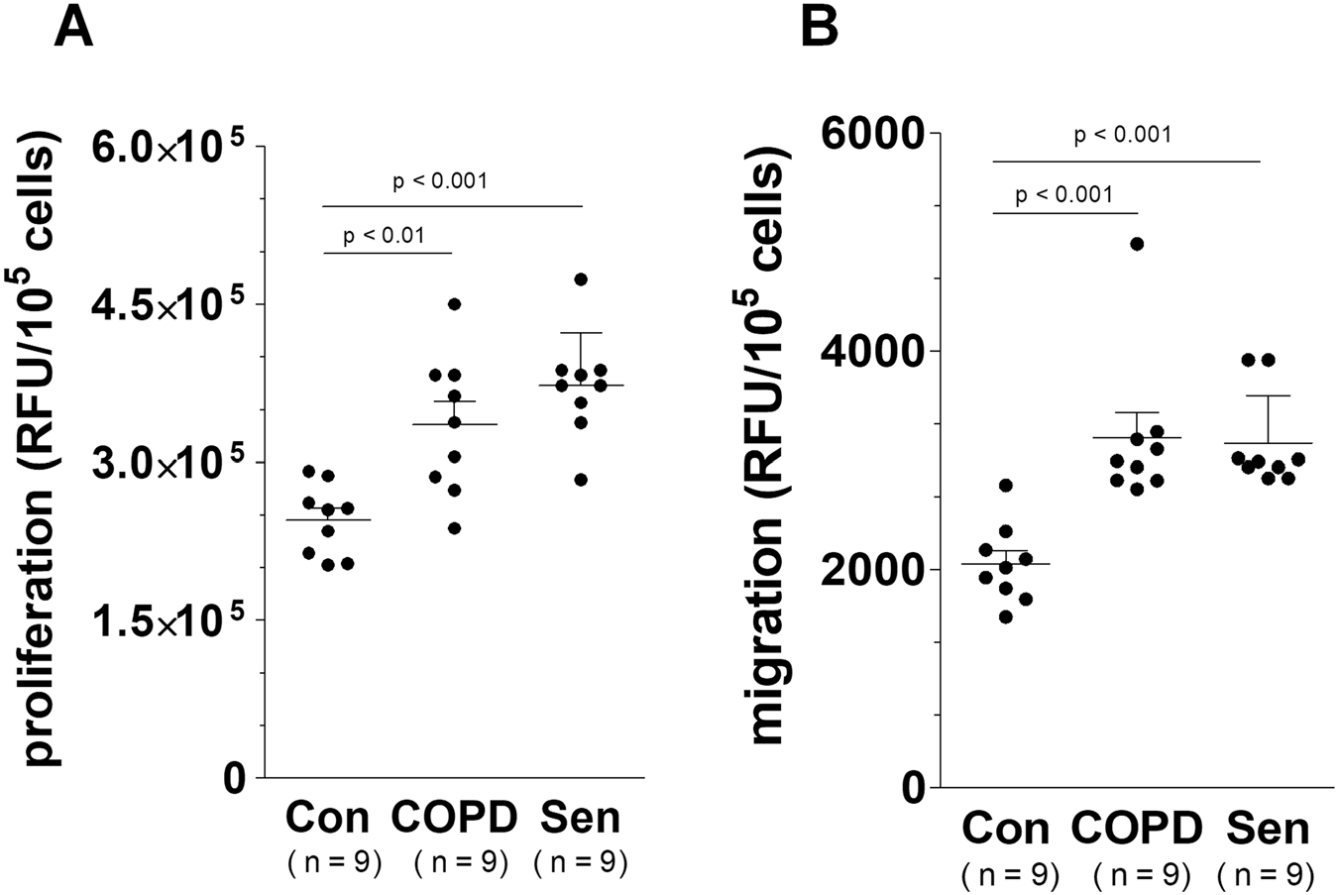
Effect of conditioned medium generated by epithelial cells subjected to serum from COPD patients and healthy volunteers, and by replicatively senescent epithelial cells (Sen) on proliferation (**A**) and migration (**B**) of A549 lung cancer cells. The results are expressed as means ± SEMs. RFU: relative fluorescence units.

In the final part of our study, we determined whether the senescence-like phenotype that HBECs developed in response to serum from COPD patients corresponds to an intensified progression of lung cancer cells. For the model, we used a line of non-small cell lung carcinoma cells A549, which had already been used by our group in research into the pathogenesis of COPD-related lung cancer^13^. Experiments showed that HBECs exposed to serum from COPD patients promote various steps of the cancer cell progression, including adhesion (an effect associated with direct cell-cell contact), proliferation and migration (effects mediated by soluble agents released by epithelial cells) (Fig. 8A,B,C). This observation, which suggests two modes of procancerogenic activity by senescent cells agrees with previous findings obtained for senescent peritoneal mesothelial cells and their action on ovarian cancer, and on senescent fibroblasts stimulating breast cancer cells^37^. An involvement of senescence in the pro-cancerous activity of conditioned medium generated by epithelial cells subjected to serum from COPD patients was eventually confirmed in experiments in which effects exerted by these cells on proliferation and migration of lung cancer cells were compared with those caused by epithelial cells that senesced replicatively due to an exhaustion of their ability to divide. Results of these measurements revealed that the stimulation of both elements of cancer cell progression in these groups was identical and significantly higher than in the control cells (Fig. 9A,B).

## Conclusions

All in all, this paper confirms that serum from COPD patients can alter the properties of bronchial epithelial cells, in particular to induce a senescence-related phenotype. Furthermore, these prematurely senescent cells have a great potential to stimulate various aspects of lung cancer cell progression, including adhesion, proliferation, and migration. These observations justify the conclusion that COPD may contribute to the development of lung cancer through the induction of cellular senescence in the normal bronchial epithelium. In this context, further detailed experiments are needed to delineate the factors present in serum from COPD patients that are capable of inducing premature senescence in bronchial epithelial cells.

## Data Availability

All data analyzed during this study are included in this published article.
